# The microbial landscape of tumors: a deep dive into intratumoral microbiota

**DOI:** 10.3389/fmicb.2025.1542142

**Published:** 2025-05-20

**Authors:** Sajjad Asgharzadeh, Maryam Pourhajibagher, Abbas Bahador

**Affiliations:** ^1^Department of Microbiology, School of Medicine, Tehran University of Medical Sciences, Tehran, Iran; ^2^Dental Research Center, Dentistry Research Institute, Tehran University of Medical Sciences, Tehran, Iran

**Keywords:** microbiome, intratumoral microbiota, detection, cancer development, anticancer therapy

## Abstract

Microorganisms in the human body play crucial roles in various health and disease processes. Research indicates that diverse bacterial species are implicated in numerous cancer types. Apart from its involvement in cancer initiation and progression, the microbiome holds promise as a biomarker for diagnosing cancer, assessing risk, and determining prognosis. Intratumoral microbes profoundly impact tumor biology by regulating the initiation and progression of tumors and modulating their response to chemotherapy, radiotherapy, and immunotherapy. A deeper understanding of the role of the intratumoral microbiome in cancer requires further investigation into its effects and underlying mechanisms. This review delves into the significance of intratumoral bacteria in cancer initiation, progression, and metastasis, their impact on cancer treatment outcomes, and Approaches Employed for Profiling the Intratumoral Microbiome.

## 1 Introduction

The human body consists of 21 × 10^13^ (women) to 30 × 10^13^ (men) eukaryotic cells, alongside numerous microorganisms engaged in symbiosis, commensalism, and parasitism. These interactions shape coevolution and vary with external factors and host conditions (Sender et al., 2016; Altveş et al., 2020; Drew et al., 2021). The microbiota includes microorganisms in the skin, oral cavity, respiratory, gastrointestinal, urinary, and reproductive systems, while the microbiome refers to their collective genes (Colella et al., 2023). Recent research has identified microbiota in organs once thought sterile, such as the kidney, prostate, lung, liver, pancreas, and breast. The gut alone harbors over 100 trillion bacterial cells, with microbiota- associated cells comprising ∼90% of the human body (Guinane and Cotter, 2013; Sender et al., 2016; Dekaboruah et al., 2020; Cao et al., 2024). These microorganisms play crucial roles in host physiology, particularly in the immune, metabolic, structural, and neurological systems (Adak and Khan, 2019). Moreover, microbiota imbalances are linked to various diseases, including neurological (Parkinson’s, Alzheimer’s), cardiovascular (hypertension, atherosclerosis), immune- related (allergies, autoimmunity), metabolic (obesity, diabetes), and cancer (Thursby and Juge, 2017; Colella et al., 2023). The link between microbiome and cancer has a long history. In 1886, Doyen isolated *Micrococcus neoformans* bacteria from several tumors and verified its tumorigenicity in animals (Dudgeon and Dunkley, 1907). Later, in 1911, Rouse discovered that *avian sarcoma leukosis* could be transmitted through a filter of tumor- free cell extracts and come up with cancer. As a result, he was the first person to present viruses as one of the causes of cancer (Rous, 1911). Marshall and Warren’s 1983 research provided the first evidence of bacteria’s role in cancer development. By isolating *H. pylori* from biopsy samples from the intact areas of the antral mucosa and observing this bacterium in almost all patients with active chronic gastritis, duodenal ulcers, or gastric ulcers, they proved the role of this bacterium in these diseases and gastric cancer (Marshall and Warren, 1984). Currently, research shows that different bacterial species are involved in several types of cancer, such as esophageal cancer, breast cancer, head and neck cancer, prostate cancer, and pancreatic cancer (Goodman and Gardner, 2018). Indeed, microorganisms have been estimated to be responsible for developing 15%–20% of cancers, the world’s second-leading cause of mortality (Gebrayel et al., 2022). The microbiome’s role in cancer development extends beyond direct infections, significantly impacting the tumor microenvironment (TME). The TME is a dynamic and complex network composed of fibroblasts, immune cells, vascular structures, adipocytes, pericytes, and extracellular matrix components that collectively influence tumor behavior through biochemical and mechanical interactions (Balkwill et al., 2012). Growing research underscores the microbiota as a crucial external factor affecting tumor progression via its metabolic byproducts, immune interactions, and signaling influences within the TME (Yang et al., 2023). Microbial metabolites function as bioactive compounds that regulate essential processes such as inflammation, angiogenesis, immune response, and epithelial-mesenchymal transition (EMT). For example, secondary bile acids like deoxycholic acid (DCA) shape cancer-associated fibroblasts (CAFs) into a pro-tumorigenic state by activating metabolic and signaling pathways. Likewise, lithocholic acid (LCA) influences immune regulation by modulating T-helper 17 (Th17) and regulatory T cell (Treg) differentiation, contributing to tumor immune evasion. Additionally, bacterial lipopolysaccharides (LPS) can directly affect epithelial cells, promoting EMT and triggering vascular endothelial growth factor (VEGF) signaling, which supports angiogenesis and enhances metastatic potential (Liu Y. et al., 2023; Anwer et al., 2025). Beyond metabolic effects, specific bacterial species contribute to carcinogenesis by interfering with host cell signaling mechanisms. Bacteria release toxins that disrupt cellular equilibrium, leading to genetic instability and inflammation that fosters tumor growth (Song et al., 2018). This intricate interaction between microbial elements and host pathways plays a role in tumor initiation and progression. On the other hand, microbes have also been investigated as potential cancer treatments. Over a century ago, Dr. William B. Coley observed spontaneous tumor regression in patients with *streptococcal* infections and created “Coley’s toxins,” a formulation of heat-killed bacteria that showed promise in cancer therapy. Building on these findings, *Bacillus Calmette-Guerin* (BCG) remains the only FDA-approved bacterial agent used to treat superficial, non-muscle invasive bladder cancer (NMIBC) (Kramer et al., 2018). More recently, bacteriophages have garnered attention for their ability to influence tumor growth, highlighting the microbiome’s dual role in both cancer progression and treatment (Cao et al., 2024). The microbiota exerts its impact even from distant body sites, shaping systemic immune responses that affect tumor behavior. The ability of microbial metabolites to exert both tumor-promoting and tumor-suppressing effects—depending on their context and concentration—further illustrates the intricate nature of microbiome-TME interactions. Beyond its role in carcinogenesis and cancer progression, the microbiome has emerged as a promising biomarker for cancer diagnosis, risk assessment, and prognosis (Ciernikova et al., 2022). Given its importance in diagnosing, progressing, and treating various cancers, further exploration of intratumoral microbiota characteristics and their influence on tumor growth is crucial. Additionally, advancing techniques for studying tumor-associated microbes will provide deeper insights into their therapeutic potential. Gaining a broader understanding of these systemic effects provides valuable insights into potential therapeutic strategies that leverage microbiome modulation to influence TME dynamics and ultimately manage tumor progression (Rossi et al., 2020).

This study aims to explore the characteristics of intratumoral microbiota, their interactions with the TME, and their implications for cancer progression and treatment. By investigating the complex relationship between microbes and tumors, this research seeks to uncover novel diagnostic biomarkers and therapeutic interventions that could enhance cancer management and patient outcomes.

## 2 Characteristics of the intratumoral microbiota

### 2.1 The origin of intratumoral microbiota

**FIGURE 1:**
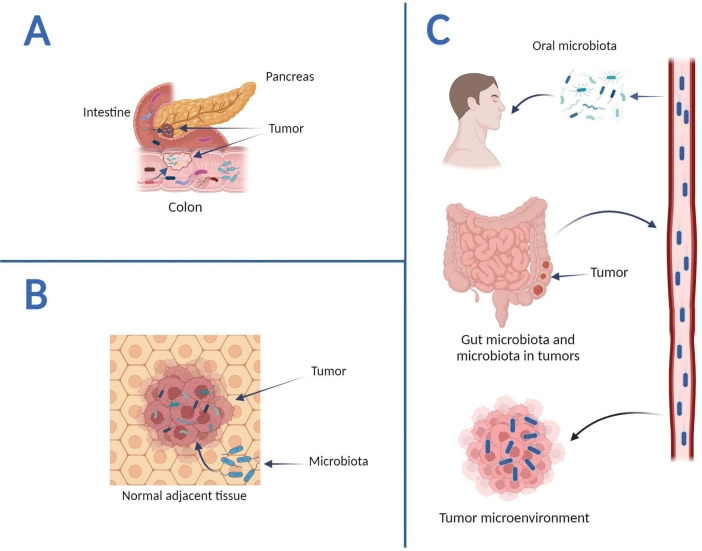
The potential origins of intratumoral microbiota. **(A)** Mucosal organs. Intestinal microbes can disrupt the mucosal barrier and access tumor sites, whereas pancreatic cancer intratumoral bacteria can penetrate tumor sites via the pancreatic duct. **(B)** Normal adjacent tissues. NATs can serve as a source of intratumoral bacteria. **(C)** The circulatory system. The hematogenous spread allows intratumor microorganisms to enter tumor sites via the mouth, intestines, tumors, and other locations—graphics created with BioRender.com.

Despite the great importance of intratumoral microbes, their origin remains unknown. According to recent studies, there are three possible origins for the intratumoral microbiome. The first way is through the mucosal barrier (Figure 1A). In this way, mucosa-colonizing microorganisms may invade the tumor through damaged mucosa. They thereby become intratumoral microbiota, which can perform complex functions. Intratumoral microbiota is typically observed in malignancies from mucosal tissues, including colorectal, pancreatic, cervical, and lung cancers (Yang et al., 2023). Although human mucosal organs harbor abundant microbiomes, the prevailing notion that intratumoral microbiota exclusively originates from the mucosal site across the mucosal barrier fails to explain the entirety of intratumoral microbial populations. Some detected intratumoral bacteria are infrequently observed in the mucosal organs associated with their respective tumors, whereas others are frequently seen in non-mucosal tumors. This indicates the possibility of alternative sources for intratumoral bacteria (Wu J. et al., 2024). The second route is the nearby normal tissue (Figure 1B) as, according to a 2020 study, the bacterial composition of normal adjacent tissues and tumor tissues is remarkably similar (Nejman et al., 2020). Similar studies expanded, and bacteria were discovered in previously thought to be sterile organs. The bacteria found in normal adjacent tissues (NATs) may have originated from TMEs, which could explain this similarity (Wu J. et al., 2024). As a result, it is still being determined if NATs are among the origins of intratumor bacteria, so additional research is needed.

The circulatory system, which encompasses blood, lymphatic fluid, and the alimentary tract’s internal passages, represents intratumoral microorganisms’ ultimate and third origin (Figure 1C). In this approach, bacteria in the mouth, intestine, and other non-sterile sites can be transported to the tumor site via the circulatory system and penetrate the tumor through damaged blood vessels. For example, *Fusobacterium nucleatum* is one of the prominent members of the human oral microbiome; these bacteria use a hematogenous route to reach colon adenocarcinoma (Abed et al., 2016). It should be noted that microbial species in the circulatory system could directly enter tumor tissues. Microbes infiltrating the bloodstream from various locations may be transferred to the TME by the necrotic cell-released debris in tumors or the chemotactic gradient. Furthermore, erythrocytes were suggested as potential transporters of bacteria to tumors (Huang et al., 2022). In general, intratumoral bacteria originate from various sources and have a solid link to the oral and intestinal microbiota. Furthermore, research suggests that bacteria enter tumors through multiple methods.

### 2.2 Diversity and differences of intratumoral microbiota

**FIGURE 2:**
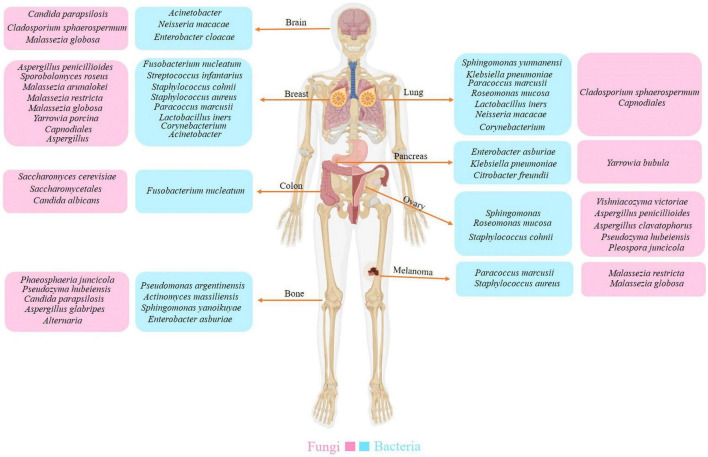
The diversity of intratumoral microbiota. Each tumor type possesses a distinct composition of bacterial and fungal species. Researchers have also identified a unique microbiota between the tumor and peritumor tissues.

Considering the potential variation in microbial origins within tumors, the microbial composition varies across different cancer types. Research on the microbiomes of seven cancer types—lung, breast, pancreatic, ovarian, brain, bone, and melanoma—has demonstrated that each tumor possesses a distinct microbiome composition (Figure 2) (Nejman et al., 2020). Recent research found DNA and fungal cells in several common human malignancies. The microbiome community compositions varied depending on the type of cancer. In addition, bacteria predominated in the tumor’s microbial populations, while fungi were scarce. Furthermore, similar community compositions were discovered in nearby normal tissues (Nejman et al., 2020; Narunsky-Haziza et al., 2022). Certain microorganisms have been found in various cancers. However, the frequency varies according to the type of cancer (Liang et al., 2023). Because cancerous tissue has less microbial diversity than normal tissue, tumors can form a unique habitat that favors specific bacterial species. Most of these bacteria are commensal species that reside primarily within intracellular compartments. The presence of heterogeneous bacterial communities in cancer tissues suggests potential multifunctional interactions with cancer cells, influencing tumor progression and microenvironment dynamics (Fu et al., 2022).

The diversity of the intratumoral microbiota introduces complexities that can significantly impede research efforts. Tumor dynamics are influenced by multiple factors, including cell proliferation, genomics, microbial interactions, and metagenomics. The intratumoral microbiota shapes the tumor microenvironment by modulating immune responses, inflammation, and metabolic patterns. Moreover, microbial composition varies across different cancer stages, further complicating the analysis of tumor-resident microbiota (Dovrolis et al., 2024). For instance, in oral squamous cell carcinoma (OSCC) and colorectal cancer (CRC), these microbial shifts dynamically influence tumor aggressiveness and immune responses. In OSCC, *Capnocytophaga*, *Fusobacterium*, and *Treponema* increase in later stages, while *Streptococcus* and *Rothia* are more abundant in precancerous stages. Advanced- stage cancer (T4) sees reduced bacterial diversity, with *Streptococcus* declining, *Rothia* disappearing, and *Capnocytophaga* becoming dominant (Singh et al., 2023). These changes impact immune activation, favoring bacteria that suppress immune responses. Similarly, in CRC, microbial composition evolves with disease progression, as *Fusobacterium nucleatum* becomes significantly enriched in advanced stages (III/IV), contributing to immune evasion and tumor progression. Early-stage CRC exhibits greater microbial diversity, with *Bacteroides* and *Prevotella* being more prevalent, whereas late-stage CRC shows reduced diversity. *Bifidobacteria* is strongly associated with signet ring cell carcinoma, a more aggressive CRC subtype, while virulence-associated bacterial genes become more abundant in advanced CRC, potentially driving metastasis. Together, these microbial shifts in OSCC and CRC underscore the crucial role of intratumoral bacteria in cancer progression and immune modulation (Xue et al., 2023). These findings underscore the inherent heterogeneity of intratumoral microbiota across patients and cancer stages, adding complexity to efforts aimed at defining standardized microbial signatures for disease chronology. Given these challenges, further studies employing tumor tissue biopsy specimens are necessary to precisely identify tumor-invading bacteria and elucidate their interactions with the intratumoral immune system. A more comprehensive understanding of the dynamic changes in microbial communities across different cancer types and stages is essential for developing targeted therapeutic and diagnostic strategies.

While intratumoral microbiota exists within the TME, its composition and distribution exhibit distinct characteristics due to selective pressures and microbial adaptation. The distribution of intratumoral microbiota varies within different tumor regions, as observed in CRC and adenoma (Kyriazi et al., 2024). Certain bacterial clusters correlate with specific tumor cell features, such as diminished p53 expression, highlighting micro-niche diversity within the TME. Tumor-associated microbial communities often differ significantly from those in adjacent healthy tissue at the phylum, order, or genus level, suggesting that tumors exert selective pressures that shape microbial composition differently from normal tissue (Lombardo et al., 2024). Intratumoral microbiota can originate from the local microbiome of tumor-bearing tissues or translocate from distant sites, such as the gut or oral cavity, via disrupted mucosal barriers or circulation (Gong et al., 2023). The broader TME is influenced by these translocating microbes, which may subsequently establish themselves within the tumor. Intratumoral bacteria directly interact with immune cells, stromal cells, and the extracellular matrix (ECM), influencing microbial composition and tumor progression. These interactions contribute to a dynamic tumor ecosystem (Xu et al., 2024).

Additionally, tumors present unique conditions such as hypoxia, acidity, nutrient competition, and immune activity, which selectively favor the survival of specific microbial species (Arneth, 2020). For example, anaerobic bacteria thrive in hypoxic regions, while acidophilic species like *Lactobacillus* adapt to the acidic tumor environment (Kyriazi et al., 2024). Understanding these variations is essential for unraveling the role of microbial communities in tumor progression and therapeutic response.

## 3 The impacts of the intratumoral microbiota on cancer development

**FIGURE 3:**
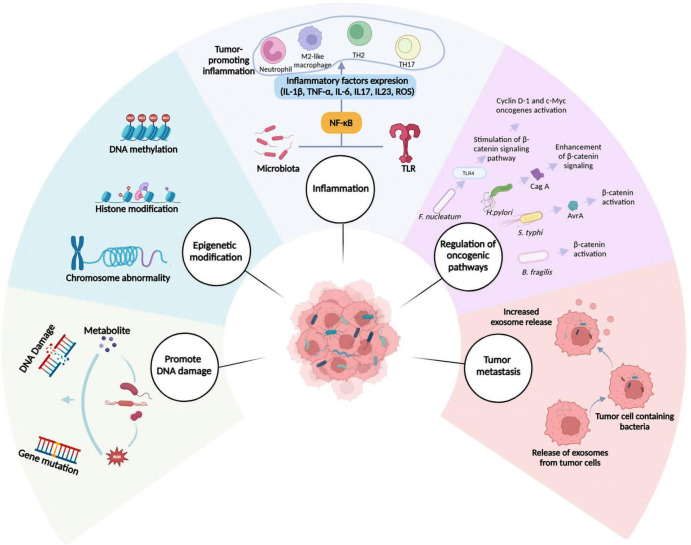
Mechanisms involved in cancer progression mediated by intratumoral microbiota. Several mechanisms have been suggested to describe the role of intratumoral microbiota in cancer initiation and progression. These include promoting DNA damage, epigenetic modifications, inflammation, regulation of oncogenic pathways, and facilitation of metastasis. NF-κB, nuclear factor kappa B; ROS, reactive oxygen species; TLR, Toll-like receptor.

Although the microbiome’s possible contribution to cancer initiation and progression is unknown, it may modify critical tumor-promoting functions in both malignant and non-malignant cells. Gaining insight into these mechanisms can enhance the effectiveness of cancer diagnosis and treatment. The subsequent section will provide an overview of the critical functions played by intratumoral bacteria in advancing carcinogenesis and development (Figure 3).

### 3.1 Promote DNA damage

Certain bacteria contain strategies for damaging DNA, which can lead to mutations and eventually cause cancer. When DNA damage surpasses the repair capacity of the host cell, it can result in apoptosis, cell death, or oncogenic transformations. In other words, DNA damage is an essential factor in carcinogenesis (Bhatt et al., 2017). Carcinogenic bacteria have developed several mechanisms to damage the host’s DNA, which include DNA-damaging molecules, proteins, and metabolites.

Such products have the potential to directly or indirectly interact with the host’s DNA, causing mutations. These metabolites include cytolethal distending toxin (CDT), colibactin, and *Bacteroides fragilis* toxin (BFT), which inflict DNA damage and induce mutations. Varieties of Gram-negative bacteria from the phylum Proteobacteria gamma and epsilon classes generate CDT. CDT is an exotoxin with unique features that enable it to be classed as both a cyclomodulin and a genotoxin. CDT is a heteromultimeric protein that consists of three subunits: CdtA, CdtB, and CdtC. Each of these subunits plays a distinct role in the overall function of CDT. CdtB is similar to DNase I in sequence homology, structure, and function and causes DNA damage (Barrett et al., 2020). CdtB exhibits its function in a dose-dependent manner. In other words, the impact of CdtB activity is influenced by the concentration or dosage of this protein. Thus, as the dose increases, the effect shifts from inducing single-stranded DNA breaks to double-stranded ones (Fedor et al., 2013). Abnormal responses to DNA damage can cause genomic instability and induce cancer. Certain *E. coli* strains contain genomic islands known as “pks islands,” biosynthetic gene clusters. This gene cluster encodes a hybrid of non-ribosomal peptide synthase (NRPS), polyketide synthase (PKS), and colibactin (Miyasaka et al., 2024). Colibactin can induce DNA double-strand breaks (DSBs), increasing genome instability and mutation rates. Colibactin exhibits genotoxic effects on the DNA of the infected host cells and the bacteria that synthesize it. Bacteria have devised various strategies to protect DNA from the effects of colibactin, including efflux and the ClbS hydrolase enzyme (Chagneau et al., 2022). BFT has been linked to diarrhea, inflammatory bowel illness, and colon cancer in various studies (Jawara et al., 2018). In a mouse model of colon cancer, pks + *E. coli* was discovered to have a synergistic effect with enterotoxigenic bacteria *Bacteroides fragilis* (ETBF), causing DNA damage to colon epithelial cells and increasing the possibility of cancer formation (Dejea et al., 2018).

Bacterial metabolites can have an indirect genotoxic effect by producing free radicals and reactive oxygen species (ROS). For example, *Enterococcus faecalis*, a commensal bacterium in the human gastrointestinal tract, can generate substantial amounts of extracellular superoxide (O2) and reactive oxygen species such as H2O2 and hydroxyl radicals through the autoxidation of membrane-bound dimethyl menaquinone (Huycke et al., 2002). These oxidants may cause chromosomal instability (CIN) and contribute to developing colorectal cancer and adenomatous polyps.

### 3.2 Epigenetic modification

In mammals, epigenetic mechanisms are critical for developing and maintaining tissue-specific gene expression patterns. Chromatin comprises nucleosome repeating units, and epigenetic mechanisms can alter chromatin structure (Ilango et al., 2020). Mammalian cells can modify their transcriptional program to environmental stimulation through epigenetic alterations, which enable them to change gene expression without altering the genetic code (Woo and Alenghat, 2022). The negative aspect is that epigenetic pathways can play an important role in oncogenesis by incorrectly inhibiting tumor suppressor genes (TSG) and activating oncogenes. Several bacteria can survive, proliferate, and evade the host’s immune system by manipulating the host’s epigenome (Cao et al., 2024). Epigenetic alterations, including DNA methylation, histone modifications, miRNA-mediated regulation, and chromatin remodeling, are commonly observed in numerous malignancies, including colorectal cancer (Wang et al., 2017). Moreover, infection with *H. pylori* can result in aberrant DNA methylation, elevating the susceptibility to gastric cancer (GC) (Liu D. et al., 2023). Histone proteins can undergo various post-translational modifications, including methylation, acetylation, phosphorylation, and ubiquitination. Among these, histone acetylation has garnered significant attention in microbiological studies of multiple malignancies, particularly breast cancer (Wu et al., 2022). Microorganisms engage in the synthesis and metabolism of diverse chemicals, which serve as epigenetic substrates and cofactors or modulate the activity of epigenetic enzymes. These interactions indirectly influence host epigenetic modifications. For example, DNA and histone methylation primarily rely on substrates such as folate and other B vitamins. Folate is an essential element of commensal intestinal microorganisms, such as the probiotic species *Bifidobacterium* and *Lactobacillus*. It participates in one-carbon metabolism, generating S-adenosylmethionine (SAM), a necessary substrate for DNA and histone methylation. Another major category of epigenetically linked compounds is short-chain fatty acids (SCFAs), which are produced by commensal microorganisms through the fermentation of indigestible complex carbohydrates and fiber (Woo and Alenghat, 2022). SCFAs modulate genomic epigenetic changes by affecting the functions of histone acetyltransferases and deacetylases. Recently, an investigation discovered a link between microbiota modification and miRNA expression in several forms of cancer. For example, In addition to targeting nucleic acid sequences, miR-515-5p and miR-1226-5p can enhance the growth of *Fusobacterium nucleatum* and *E. coli* (Liu et al., 2016).

Extensive research has indicated that intratumoral microorganisms can directly or indirectly affect host epigenetic modifications, such as DNA, histone, RNA modifications, and non-coding RNA alterations. Nevertheless, the precise molecular mechanisms underlying these epigenetic modifications induced by intratumoral bacteria require further investigation.

### 3.3 Inflammation

Inflammation is closely related to all stages of cancer development and malignant progression, as well as the efficacy of anticancer therapy. Acute inflammation triggers cancer cell death by activating an antitumor immune response, but chronic inflammation promotes treatment resistance and cancer development. Chronic inflammation can lead to immunosuppression, which results in a favorable microenvironment for carcinogenesis, tumor progression, and metastasis (Zhao et al., 2021). Intratumoral bacteria can stimulate inflammatory pathways by interacting with pattern recognition receptors within the tumor microenvironment, such as Toll-like receptors (TLRS). For example, TLR4 in non-small-cell lung cancer cells can be activated by gram-negative bacteria, which promotes tumor growth and metastasis (Sun et al., 2018). TLRs are vital in connecting innate and adaptive immunity by regulating the activation of antigen-presenting cells and essential cytokines (Duan et al., 2022). *F. nucleatum* interacts with TLRs in the tumor microenvironment and activates the TLR4/MYD88/NF-κB signaling pathway. Stimulating this pathway promotes a pro-inflammatory microenvironment that is desirable for the survival of colorectal cancer cells while preventing apoptosis. This produces a positive feedback cycle that triggers pro-inflammatory responses and accelerates the advancement of CRC (Wu Y. et al., 2024). In addition to *F. nucleatum*, certain strains of *B. fragilis* and *E. coli* can produce pro-inflammatory responses. These responses stimulate the recruitment of immune cells, such as neutrophils and MDSCs, to the tumor site. These cells are a double-edged sword since their interactions with bacteria and the host can either promote or hinder tumor formation (Scott et al., 2022).

Inflammation occurs when the immune system responds to harmful stimuli, such as pathogens, damaged cells, toxic substances, or exposure to radiation. It eliminates these harmful stimuli and initiates healing, acting as a crucial defense mechanism for maintaining health (Chen et al., 2018). However, uncontrolled acute inflammation may progress to chronic, resulting in various chronic inflammatory illnesses. The chronic inflammatory microenvironment in cancer may progress into an immunosuppressive microenvironment, promoting tumor development and inhibiting the antitumor immune response. In addition, inflammatory cells can generate ROS, a mediator of DNA damage induction (Shalapour and Karin, 2019).

### 3.4 Regulation of oncogenic pathways

Microbes can foster tumor growth via regulating oncogenes and pathways, including Wnt/β- catenin and Notch (Parida et al., 2021). β-Catenin is a versatile protein that plays an essential role in physiological homeostasis. Abnormally high expression of β-Catenin causes a variety of illnesses, including cancer. It serves as both a transcriptional co-regulator and an intracellular adhesion adaptor protein. Wnt is the primary regulator of β-catenin, a family of 19 glycoproteins that control both the β- catenin-dependent (canonical Wnt) and catenin-independent (non-canonical Wnt) signaling pathways (Shang et al., 2017). *F. nucleatum* triggers the β-catenin signaling pathway through Toll-like receptor 4. When the β-catenin pathway is engaged, it can activate downstream oncogenes, including cyclin D-1 and c-Myc, which promotes cancer growth (Chen et al., 2017). *H. pylori* produces CagA, which promotes β-catenin signaling and leads to gastric cancer (Abreu and Peek, 2014). Certain *S. typhi* strains secrete AvrA, which activates β-catenin and is linked to hepatobiliary cancer (Lu et al., 2014).

Beyond the Wnt/β-catenin pathway, microorganisms can potentially activate additional cancer- related signaling pathways. For example, *B. fragilis* activated the Notch1 and β-catenin pathways, leading to breast tissue carcinogenesis and progression (Parida et al., 2021). The JAK-STAT pathway is critically involved in colorectal cancer and other malignancies, where it often becomes abnormally activated. ETBF can trigger STAT3 activation in colorectal tumors via phosphorylation and subsequent nuclear translocation (Purcell et al., 2022).

### 3.5 Tumor metastasis

Although the exact mechanisms by which intratumoral bacteria influence tumor metastasis are not yet fully understood, recent evidence suggests that these microorganisms may play a role in initiating tumor metastasis. Microorganisms within various tumor types can contribute to tumor initiation, growth, and metastasis by influencing multiple signaling pathways (Yang et al., 2023). Exosomes, which are released by infected cancer cells, could represent another mechanism. Exosomes, additionally known as extracellular vehicles (EVs), are typically 40–100 nm membrane structures. These are secreted into fluids by various types of human body cells and contain protein, mRNA, miRNA, and signaling molecules. Exosomes play a crucial role in facilitating the transfer of proteins and RNA between cells, and from an immunological perspective, they demonstrate the capacity to present antigens (Chen et al., 2019). Tumor-derived exosomes (TEXs) have emerged as significant components originating from tumors involved in the metastatic process. Evidence indicates that TEXs can engage with host immune, epithelial, and tumor cells. Through these interactions, TEXs can modify and reprogram host cells, ultimately promoting tumor progression and facilitating cancer metastasis (Chen et al., 2021). Research findings indicate that when tumor cells are infected with bacteria, they tend to release more exosomes (Guo S. et al., 2020).

Furthermore, intracellular bacteria within tumors significantly enhance the survival of tumor cells under mechanical pressure during blood circulation. Due to fluid shear stress, cancer cells entering the bloodstream frequently undergo apoptosis during metastasis. Tumor cells harboring bacteria exhibit increased viability compared to those without, likely because intracellular bacteria modulate the cellular stress response (Koyama and Inamura, 2023).

## 4 Impact of intratumoral microbiota on anticancer therapy

The primary anticancer therapies encompass radiotherapy, chemotherapy, and immunotherapy, each employing distinct mechanisms to combat tumor growth and progression. Radiotherapy leverages ionizing radiation to damage cancer cell DNA, while chemotherapy employs cytotoxic agents to inhibit cell division. Immunotherapy, in contrast, harnesses the body’s immune system to identify and destroy malignant cells, offering a targeted approach to treatment.

### 4.1 Chemotherapy and the Microbiome

Chemotherapy is administered through genotoxic substances that damage the DNA of current tumor cells and inhibit the creation of new DNA during cell division (Salehan and Morse, 2013). The microbiome has diverse enzymatic capabilities that affect chemotherapy response and toxicity. Intratumoral bacteria’s inherent enzymes alter the effectiveness of chemotherapeutic medications through a process known as biotransformation (Lehouritis et al., 2015). Research has demonstrated that the gut microbiota affects cancer chemotherapy, especially treatments involving cyclophosphamide (CTX) and oxaliplatin. CTX’s anticancer actions are primarily due to the stimulation of antitumor immune responses via multiple immunological pathways, which assist Th1 and Th17 cells in regulating cancer proliferation. Previous investigations have suggested that the administration of cyclophosphamide can lead to alterations in the gut microbiota composition, resulting in the migration of specific gram-positive bacteria to secondary lymphoid organs. This triggers the generation of pathogenic T helper 17 (pTh17) cells and boosts the host immune system’s response driven by memory T helper 1 (Th1) cells (Viaud et al., 2013). According to studies, oral administration of *Enterococcus hirae* can restore CTX-mediated anticancer effects; thus, *Enterococcus hirae* is recognized as a valuable oncomicrobiotic (Daillère et al., 2016).

Oxaliplatin is a platinum-based antineoplastic medication. It is used in various conditions, such as neuroendocrine tumors, esophageal and gastric cancers, and advanced pancreatic cancer. This drug’s mechanism of action involves DNA damage, which induces death in cancer cells (Chambers and Illingworth, 2023).

According to recent research, *F. nucleatum*, which lives in the gut, can promote resistance to cytotoxic chemotherapy drugs coupled with oxaliplatin and capecitabine in colorectal cancer patients (Yu et al., 2017). Gemcitabine, a nucleoside analog, is frequently used to treat pancreatic, lung, breast, and bladder cancers. In pancreatic ductal adenocarcinoma (PDAC) tissues, the predominant expression of the long isoform of the bacterial enzyme cytidine deaminase (CDDL) is mainly attributed to Gammaproteobacteria, one of the most prevalent species in these tissues. Intratumoral bacteria expressing CDDL have been discovered to metabolize gemcitabine passively, leading to tumor resistance against this chemotherapy drug. In mouse models of colon cancer, the development of chemoresistance to gemcitabine can be counteracted by administering ciprofloxacin (Geller et al., 2017). As a result, “pharmacomicrobiomics” is gaining prominence as a new field within chemotherapy research.

### 4.2 Radiotherapy and the microbiome

Radiotherapy is a significant curative treatment method for uncomplicated loco-regional tumors and is incorporated into at least two-thirds of cancer treatment protocols in Western nations (Chen and Kuo, 2017). Radiotherapy operates on two fundamental principles: Firstly, it directly destroys cancer cell DNA using ionizing radiation to eliminate the cancer cells. Secondly, it indirectly targets cancer cells by inducing damage to DNA through reactive oxygen species (Petroni et al., 2022). Radiotherapy (RT) targets cancer cells and can adversely affect healthy tissues and the body’s commensal microorganisms, particularly gut ones. Radiotherapy and the gut microbiota have a reciprocal impact on each other. A common side effect of radiotherapy is dysbiosis of the gut microbiota. This condition is typically marked by a reduction in beneficial microbes, such as *Bifidobacterium*, and an increase in harmful microorganisms like *Fusobacteria* and *Proteobacteria*. These changes in the gut microbiota composition exacerbate radiation-related complications, such as radiation enteropathy. Nevertheless, certain commensal microbes play a crucial role in enhancing the effectiveness of radiotherapy and mitigating adverse events associated with it. Recent research has revealed intestinal fungus modulates antitumor immune responses following radiation therapy in mice breast cancer and melanoma models. In contrast, bacteria have the opposite role and increase the response rate (Oh et al., 2021; Shiao et al., 2021). Another study revealed that radiation therapy-induced side effects, including fatigue, nausea, vomiting, and diarrhea, can potentially be mitigated by probiotics like *Lachnospiraceae* and *Enterococcaceae*. These probiotics may help reduce radiation-related damage by modulating the gut microbiome (Guo H. et al., 2020).

While direct evidence regarding the microbiome’s impact on radiation therapy efficacy remains limited, the link between radiation therapy side effects and the gut microbiome suggests the potential to adjust the gut microbiome composition to mitigate radiation-related toxicity. Such modulation could enhance the prognosis for patients undergoing radiation therapy. Future research may reveal the exact mechanisms linking the host microbiome to radiation therapy’s response and side effects. Therefore, the interplay between gut microbes, tumors, and radiotherapy is intricate, offering a vast area for research.

### 4.3 Immunotherapy and the microbiome

Immunotherapy has demonstrated promising results in recent years, introducing novel approaches for the clinical management of cancer alongside traditional treatments like chemotherapy and radiotherapy. Antitumor immunotherapies promote the host immune system’s ability to identify and eliminate cancerous cells (Iwai et al., 2017). Within the realm of immunotherapy, two significant approaches can be highlighted. First, immune checkpoint blockade specifically targets molecules such as CTLA-4 and PD-1. Second, adoptive T-cell therapy is exemplified by CAR-T therapy (Waldman et al., 2020).

Despite the remarkable effectiveness of immunotherapy, a significant number of patients do not exhibit a response. More troubling, some patients who initially exhibit promising responses to immunotherapy subsequently develop resistance (Bai et al., 2020). Significantly, emerging evidence suggests that intratumoral bacteria can affect the effectiveness of immunotherapy. Consequently, numerous studies are exploring modulating the microbiome for therapeutic benefits. Approaches include Fetal microbiota transplantation (FMT), the utilization of probiotics, and the targeted use of antibiotics (Sevcikova et al., 2022). For instance, one study found that *Clostridium* was more abundant in the melanomas of patients who responded to immune checkpoint inhibition, whereas *Gardnerella vaginalis* was more common in non-responders (Nejman et al., 2020). In another study, researchers discovered that increasing the levels of *Bacteroides fragilis*, *Burkholderia cepacia*, and *Faecalibacterium* in the gastrointestinal tract of patients receiving CTLA-4-based immunotherapy enhanced the therapeutic effect and reduced adverse side effects, such as colitis (Miller and Carson, 2020).

Considering the practicality of collecting stool samples from human donors, using FMT in immunotherapy is undeniably promising. Clinical trials have demonstrated that FMT can positively impact melanoma patients, offering potential benefits. In these studies, FMT resulted in a higher abundance of bacterial species previously linked to positive responses to anti-PD-1 therapy, enhanced activation of CD8^+^ T cells, and a reduced presence of interleukin-8-expressing myeloid cells (Davar et al., 2021). Despite its therapeutic potential, FMT is not without risks. While generally considered safe, most short-term adverse effects—such as transient diarrhea, abdominal discomfort, bloating, and constipation—are mild and self-limiting. However, the transfer of live microorganisms presents a more significant concern, particularly for immunocompromised individuals (Baxter and Colville, 2016). Although studies suggest that FMT is well-tolerated even in high-risk groups, rare but severe complications have been reported. Cases of bloodstream infections linked to extended-spectrum beta-lactamase (ESBL)-producing *E. coli* and enteropathogenic *E. coli* (EPEC) highlight the critical importance of rigorous donor screening and continual reassessment of safety protocols (DeFilipp et al., 2019). Additionally, concerns extend beyond infectious risks, as FMT may theoretically influence non-infectious conditions such as metabolic disorders, neuropsychiatric conditions, and even cancer. While long-term data have not shown significant safety concerns, ongoing surveillance, including initiatives like the FMT National Registry, remains essential to fully understanding the risks associated with this emerging therapy (Yadegar et al., 2024).

Furthermore, integrating immunotherapy with probiotic supplementation represents a promising avenue for research. This approach strategically combines one or more beneficial microorganisms into a unified formula. In a study, researchers discovered that combining Probiotic supplementation with OncoTherad had several effects. It controlled weight loss, activated the canonical TLR2/TLR4 signaling pathway (MyD88-dependent), diminished the non-canonical (TRIF-dependent) signaling pathway, suppressed the proliferative pathway driven by Ki-67 and the KRAS oncogene, and enhanced the production of IL-10 and TGF-β cytokines (Reis et al., 2022). Furthermore, research has indicated that non-targeted use of commercially available probiotics might not enhance the effectiveness of immunotherapy and could potentially lead to immunotherapy-related autoimmune reactions. In other words, improper probiotic use has been associated with a range of diseases, including inflammatory bowel diseases (IBD), celiac disease, type 1 diabetes (insulin-dependent), neurological and mental disorders, rheumatic conditions, obesity, cardiovascular issues, atherosclerosis, allergies, and cancer (Tlaskalová-Hogenová et al., 2011). Thus, while probiotics have potential benefits in cancer therapy, careful patient selection, strain-specific assessments, and controlled administration are crucial to minimizing the risks of adverse effects. Additional research is needed to reveal the underlying mechanisms of these microbial interventions and develop personalized probiotics customized for patients with diverse living environments and dietary habits. While current studies primarily investigate gut microbes, there is limited research on the impact of tumor microbes on immunotherapy effectiveness. The existence of communication between gut and intratumoral microbes remains uncertain, as does the potential influence of modifying gut microbes on intratumoral microbial composition and the host immune microenvironment. These areas warrant further exploration.

## 5 Approaches employed for profiling the intratumoral microbiome

**FIGURE 4:**
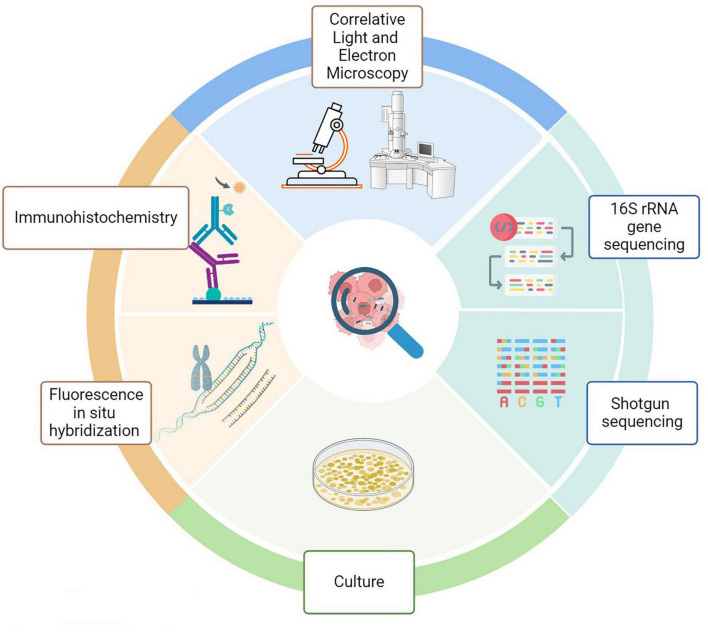
Methods for analyzing intratumoral microbiota. Technical approaches for assessing tumor- associated microbiota include molecular biology, microbiology, and histopathology. Each color reflects a particular discipline (green, molecular biology; pale green, culture; blue. microscopy; orange, histology).

Recent technological advancements have challenged the traditional belief that internal organs remain sterile in healthy individuals. When examining tumor-associated microbiota, the technical approaches closely resemble conventional microbiology methods, including culturomics and culture-independent microbial techniques (Figure 4). Together, these techniques have increased detection sensitivity and specificity. However, it is critical to recognize some restrictions.

### 5.1 16S rRNA gene sequencing

16S rRNA sequencing is a fast and cost-effective method for identifying bacteria. However, its main limitation is that it applies only to bacteria, as viruses and parasites lack the 16S rRNA gene (Cénit et al., 2014). This gene (∼1,500 base pairs) consists of conserved regions interspersed with nine variable regions (V1–V9) (Abellan-Schneyder et al., 2021). Among them, the V4 region is preferred for bacterial diversity analysis due to its high specificity and rich data content (Lane et al., 1985). A study using this method to profile oral microbiota at different cancer stages revealed significant shifts in bacterial composition from precancerous to advanced stages. The data also highlighted interactions between the microbiota and the tumor immune system, suggesting an immunosuppressive and non-immunogenic tumor environment (Singh et al., 2023).

### 5.2 Shotgun sequencing

Shotgun sequencing is a powerful method for analyzing the entire genetic material of a microbial community without relying on PCR amplification (Wiseschart et al., 2019). This approach is widely used in metagenomics and can identify bacteria down to the species level and analyze viromes, which lack a universal marker for identification. Studies using shotgun sequencing have shown that the microbiome of soft tissue sarcomas holds prognostic significance, with viral abundance linked to NK cell infiltration and cancer outcomes (Perry et al., 2023). Additionally, research indicates that this method provides a more comprehensive view of the gut microbiota than 16S rRNA sequencing (Durazzi et al., 2021).

### 5.3 FISH

Fluorescence in situ hybridization (FISH) is a molecular cytogenetic technique that uses fluorescent probes to hybridize with specific nucleic acid sequences (Decordier and Kirsch-Volders, 2013). This method offers several advantages, including rapid results (within 60–90 min), identification at both genus and species levels, detection of extracellular microorganisms, minimal equipment requirements, and accurate diagnosis of difficult-to-identify bacterial pathogens. Additionally, it can detect antimicrobial resistance mutations in ribosomal RNA genes (Frickmann et al., 2017). However, FISH has limitations, such as requiring highly skilled personnel, lower sensitivity compared to PCR for primary samples, and the necessity for targeted probe design (Smolina et al., 2010; Gu et al., 2022). It is commonly used for microbial detection and studying microbial interactions. Research using FISH in a mouse model of hepatocellular carcinoma (HCC) demonstrated significant differences in intratumoral bacterial composition between tumor and adjacent non-tumor tissues (Xue et al., 2024).

### 5.4 IHC

Immunohistochemistry (IHC) is a vital technique for pathologists, enabling the precise localization and quantification of specific molecules within tissues through antigen-antibody interactions. It significantly identifies disease-related molecules and evaluates predictive and prognostic biomarkers in malignancies (Magaki et al., 2019). A key advantage of IHC is that it preserves the histological structure, allowing for the assessment of molecular expression within the tissue microenvironment (Kim et al., 2016). In microbial research, antibodies targeting bacterial LPS and lipoteichoic acid (LTA) are commonly used to detect Gram-negative and Gram-positive bacteria (Xue et al., 2023). Studies utilizing IHC have shown that *Bacteroides* and *Blautia* at tumor sites correlate with improved prognosis in patients with poorly differentiated colorectal cancer (CRC), suggesting their potential as prognostic biomarkers (Zhao et al., 2023). This suggests that these intestinal bacteria could be biomarkers for predicting poorly differentiated CRC prognosis.

### 5.5 CLEM

Correlative Light and Electron Microscopy (CLEM) is a powerful technique that combines the advantages of light and electron microscopy, enabling precise identification and high-resolution imaging of cells and molecules (de Boer et al., 2015). It is widely used in biological research to study intracellular structures, cellular dynamics, and tissue organization (Cognigni et al., 2023). CLEM has also been applied in neuroscience and cancer research, particularly for investigating intratumoral microbiota. Studies utilizing CLEM have confirmed bacterial infiltration into melanoma cells, including *F. nucleatum, Actinomyces odontolyticus*, and *Staphylococcus caprae* (Kalaora et al., 2021). Additionally, it has demonstrated the intracellular presence of bacteria in breast cancer, lung cancer, melanoma, and glioblastoma (Nejman et al., 2020). This advanced imaging method provides crucial insights into microorganism-cancer interactions, contributing to developing targeted therapeutic strategies (Xie et al., 2022).

### 5.6 Culture

Culturing microorganisms remains a valuable method for studying intratumoral microbiota, despite the challenges posed by the unique growth requirements of different microbes (Lagier et al., 2018). While metagenomic studies generate vast amounts of data, their outcomes are often limited by study design or data analysis flaws (Bilen et al., 2018). Culturing provides critical insights for both in vitro and in vivo experiments. However, many microbes have specific growth needs, such as anaerobic conditions or host cell systems, making their cultivation difficult (Bilen et al., 2018). Recent advancements in culturomics—a high-throughput approach combining diverse culture conditions with bacterial identification—have facilitated the cultivation of previously unculturable microbes (Lagier et al., 2012; Lagier et al., 2018). Although culturomics has significantly expanded knowledge of gut microbiota, its application in intratumoral microbiota research remains limited due to the low biomass of these microbes in tumor tissues (Xie et al., 2022).

## 6 Conclusion

Intratumoral bacteria play a pivotal role in the microecology of tumors, influencing cancer progression, response to therapies, and potential treatment outcomes. Exploring tumor-associated microbiota is an emerging field, progressively broadening our understanding of microbial contributions to cancer biology. These contributions include promoting DNA damage, epigenetic modifications, inflammation, oncogenic pathway regulation, and metastasis facilitation. Moreover, intratumoral microbiota offers a promising frontier for innovative therapeutic approaches, including biomarker-based diagnostics and adjunctive therapies.

Future research should focus on translating these insights into clinical applications, such as personalized microbiota-based therapies tailored to individual tumor profiles and immune microenvironments. This could involve microbiome editing strategies, including precision probiotics or engineered bacterial strains, to enhance therapeutic efficacy while minimizing adverse effects. Additionally, the integration of machine learning in microbiome analysis holds immense potential to decipher complex microbial interactions and predict therapy outcomes. Advanced computational tools could refine diagnostics, identify microbial signatures of treatment response, and guide the development of personalized interventions. The field is poised to transform our approach to cancer diagnosis and treatment by addressing these promising areas, paving the way for more effective and individualized oncology care.
